# Collaboration gets the most out of software

**DOI:** 10.7554/eLife.01456

**Published:** 2013-09-10

**Authors:** Andrew Morin, Ben Eisenbraun, Jason Key, Paul C Sanschagrin, Michael A Timony, Michelle Ottaviano, Piotr Sliz

**Affiliations:** SBGrid Consortium and the Department of Biological Chemistry and Molecular Pharmacology, Harvard Medical School, Boston, United States; SBGrid Consortium and the Department of Biological Chemistry and Molecular Pharmacology, Harvard Medical School, Boston, United States; SBGrid Consortium and the Department of Biological Chemistry and Molecular Pharmacology, Harvard Medical School, Boston, United States; SBGrid Consortium and the Department of Biological Chemistry and Molecular Pharmacology, Harvard Medical School, Boston, United States; SBGrid Consortium and the Department of Biological Chemistry and Molecular Pharmacology, Harvard Medical School, Boston, United States; SBGrid Consortium and the Department of Biological Chemistry and Molecular Pharmacology, Harvard Medical School, Boston, United States; SBGrid Consortium and the Department of Biological Chemistry and Molecular Pharmacology, Harvard Medical School, Boston, United Statespiotr_sliz@hms.harvard.edu

**Keywords:** Cutting edge, research computing, computational tools and techniques, software

## Abstract

By centralizing many of the tasks associated with the upkeep of scientific software, SBGrid allows researchers to spend more of their time on research.

Scientists increasingly rely on computational tools and techniques to collect, analyse and interpret experimental data and results (Foster, 2006) and they might use dozens of different computational tools in the course of a single project (Anderson et al., 2007). Many of the most useful programs have been developed by other scientists (Hannay et al., 2009; Morin and Sliz, 2013), but these scientists often have little or no time to support the widespread distribution of their software and/or to provide the support needed to install it on a variety of different hardware devices and operating systems. Consequently, as our reliance on software developed by other scientists increases, so do the costs and burdens of supporting this software. However, if these costs and burdens can be shared, they will fall, access to the software will increase, and new computational resources will emerge.

SBGrid (www.sbgrid.org) is a collaboration established in 2000 to provide the structural biology community with support for research computing. Such collaborations have traditionally been supported by public funding agencies (Finholt, 2003). However, SBGrid is unique in that its ongoing operations are funded exclusively by its members. By sharing the costs of research computing support across many research groups, SBGrid achieves efficiencies through economies of scale, the sharing of expertise and cooperation to promote common goals.

The primary service offered by SBGrid is the collection, deployment and maintenance of a comprehensive set of software and computational tools that are useful in structural biology research. The SBGrid software library is effectively a kind of scientific ‘app store’ that allows users to access a wide range of up-to-date applications without having to download, compile, configure, maintain or update software. Moreover, the SBGrid model holds the potential to ease the burdens and costs of providing support for research computing in any area of science that is reliant on computational tools and techniques, thus freeing up more time and resources for actual research.

## The benefits of the shared approach

**Figure 1.**
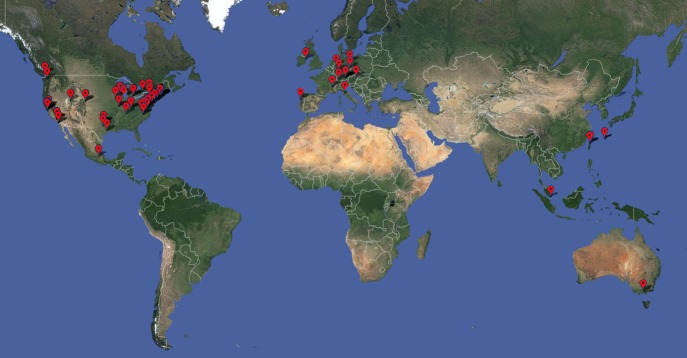
Worldwide distribution of SBGrid member laboratories as of May 2013. The SBGrid software library spans the spectrum of techniques commonly utilized by structural biologists, including X-ray crystallography, electron microscopy, NMR, 2D crystallography, bioinformatics, computational chemistry, small angle scattering, tomography, modelling, visualization and structure prediction.

SBGrid began as a collaboration between a handful of structural biology laboratories in the north-eastern US to support software applications used in X-ray crystallography (see Box 1). As more laboratories joined the collaboration, SBGrid began supporting software used in other common structural biology techniques, and today offers a library of over 270 different scientific applications and software suites to more than 245 laboratories and research groups in 16 countries (see Figure 1).

Box 1. **The origins and growth of SBGrid**

SBGrid was started in 2000 by one of the present authors (Piotr Sliz) as a home-grown solution to the challenge of supporting and maintaining a few dozen X-ray crystallography applications run on SGI IRIX and Linux workstations in the laboratories of Stephen Harrison and the late Don Wiley at Harvard University and Children’s Hospital Boston, and Ya Ha and Karin Reinisch at Yale Medical School. This support included other existing collaborative software projects in X-ray crystallography such as CCP4 (Winn et al., 2011) and later PHENIX (Adams et al., 2010)—both of which are currently contributors to SBGrid.

In 2002, Sliz and Harrison relocated to Harvard Medical School, several additional research groups joined SBGrid and it initiated software support for electron microscopy (EM), nuclear magnetic resonance (NMR) and other structural biology techniques. It also began charging user-fees to recover operational costs. In 2004, in response to demand from Mac users, SBGrid re-compiled the majority of its applications to run under OS X, and today approximately 50% of members use Macs (see Box 2). In 2007, in an effort to support more computationally demanding applications, SBGrid established a Virtual Organization within the Open Science Grid.

2009 was notable as SBGrid established itself as an NIH-compliant non-profit service centre in the Department of Biological Chemistry and Molecular Pharmacology at Harvard Medical School, and developed a unified end-user license agreement (with the help of the Harvard University Office of Technology Development). The first pharmaceutical company laboratories (Genzyme, Novartis and Biogen) also joined. These commercial members are supported with a subset of applications in the SBGrid library suitably licensed for installation in for-profit laboratories. Several synchrotron beam lines also became members in 2009.

SBGrid also makes use of its position as active intermediary between software developers and structural biologists to undertake a variety of activities:

# regular seminars and webinars in which creators of popular programs can teach and interact with SBGrid member users, answer questions and demonstrate use of their applications. These sessions are also broadcast over the web and archived for later viewing.

# policy and advocacy on behalf of the wider research computing community (Morin et al., 2012a, 2012b).

# the organization of schools and workshops. To date there have been three schools/workshops in Boston and one in Heidelberg.

SBGrid actively curates the software library, identifies new and useful software and applications, configures and compiles applications, and automatically installs the collection to computer workstations in member laboratories. SBGrid also continuously updates and upgrades programs in the collection with newer versions and bug fixes as they become available. This entire process is completely automated and transparent, requiring no input or maintenance from end-users. After members join SBGrid, all of the programs in the collection are installed and updated automatically and reside locally on member laboratory computers (see Box 2).

Box 2. **Technical details**

The SBGrid software deployment and update system, internally referred to as the ‘sync system’, uses a shell script wrapper around a common file synchronization tool called rsync to install and automatically update the UNIX software environment on a large number of internet-connected machines around the world. The system uses a MySQL database to build customized download lists that determine which software titles are available to a particular laboratory. For each application the database contains public information about that software (such as its URL and links to documentation) and information that is specific to SBGrid (such as the installation directory, and information on which groups are licensed to use the software). Each member laboratory is designated as being either non-profit, government, academic or commercial, and the database contains a set of associated download credentials for each research group.

The sync system uses this information to build a default list of applications that a member laboratory can access, and the rsync daemon on the SBGrid distribution server provides authenticated downloads of the software available in these ‘include files’. Client machines that host an installation of the SBGrid software environment run a shell script every 15 min via a cron job that performs a simple check over HTTP to see if updates are available and initiates the rsync download. SBGrid can initiate global updates that are applied to every installation, and it can also initiate a single site update in response to bug reports or requests for new software titles.

The software available on the client machines is organized into a single top-level directory with subdirectories for each operating system and CPU architecture combination (e.g., i386-linux, x86_64-linux, i386-mac, etc). Each application is installed in its own directory in the next level of the file system, and in each application directory each version of the application is installed in a separate subdirectory. This allows multiple versions of a single installed application. The software environment configures a single default version, but a simple user-controlled version switching mechanism is available so that users can, for example, select an older version to check prior results or work around bugs in newer versions.

The user initializes the SBGrid software environment by sourcing a single file into their UNIX shell, using provided configuration scripts for both sh and csh syntax shells. The shell environment does a number of tests to determine system type and then sets up a customized environment for each operating system depending on CPU type and operating system release. The sync system and software environment layout is flexible enough to accommodate the wide variety of IT environments that SBGrid member laboratories operate in and has proven to be robust and scalable.

SBGrid also provides the wider structural biology community with access to supercomputing facilities across the US in collaboration with the Open Science Grid (OSG: Pordes et al., 2007) via the SBGrid Science Portal. Currently, SBGrid provides access to two grid-enabled services: the Wide-Search Molecular Replacement (WSMR; Stokes-Rees and Sliz, 2010) service for determining crystallographic phase using the Phaser program (McCoy et al., 2007), and the Deformable Elastic Network (DEN) service for refining low-resolution electron density data (O’Donovan et al., 2012; Schröder et al., 2010).

SBGrid has also developed a prototype system for storage and transmission of large experimental datasets collected at synchrotron facilities (Stokes-Rees et al., 2012), and completed a pilot project with WeNMR to optimize cyber-infrastructure utilization between European and US computing facilities (Wassenaar et al., 2012).

Finally, to support the development of cutting edge structural biology software, SBGrid operates a Developer Support Program which, among other things, offers software developers letters of support for grant applications, software beta testing in selected member laboratories and access to programming tools and resources.

**Figure 2.**
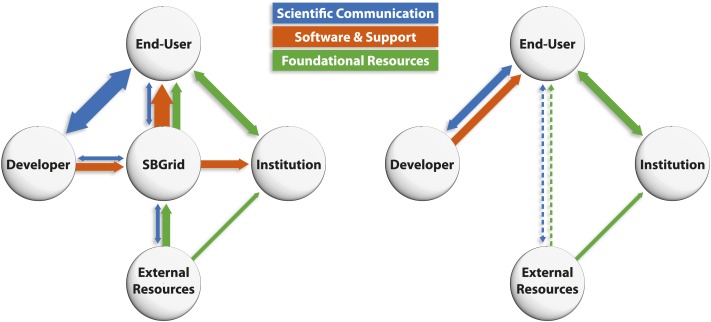
Schematic representations of the interactions between developers, end-users and institutions. (Left) By providing software and support (orange lines) to end-users and institutions, SBGrid frees up time for developers to have scientific interactions (blue lines) with the scientific community, and reduces the amount of time end-users and institutions need to spend updating and maintaining software, thus leaving more time for research. SBGrid also facilitates access to external computing resources (green lines). In the traditional model for supporting research computing (right), the burden of maintaining and updating software falls on developers and users (orange line), thus reducing the time available for other more productive activities (blue line). Moreover, access to the most powerful external computing resources is limited to a small number of computationally sophisticated end-users (dashed lines).

Collaborations like SBGrid provide benefits for the research community, software developers and institutions alike (see Figure 2). Member laboratories and end-users benefit directly from comprehensive licensing (see Box 3) and support for hundreds of software applications, each of which might otherwise demand significant time, expense and expertise to obtain, install and maintain. This centralized support means that software is maintained and updated on a regular basis, and it also reduces the problems associated with the high turnover of staff that is commonplace in academic environments. By offering access to a consistent and comprehensive set of research software, SBGrid promotes collaboration and reproducibility and, crucially, allows scientists to spend more of their time doing science.

Box 3. **Licensing model**

As part of its service to members, SBGrid consolidates licensing agreements for all software in the collection into a single end-user license agreement that users agree to when they join SBGrid. This simplifies license administration for software creators and saves end users and their institutional legal counsels from having to complete tens (or possibly hundreds) of individual license agreements.

SBGrid applications are classified into one of four categories, depending on the type of license chosen by the creator or owner of the software: (i) open source software (OSS); (ii) software that is freely available to academic members; (iii) software that is freely available to non-profit groups who are members of SBGrid; (iv) software subject to restrictions. Of the 275 software titles currently in the SBGrid library, 162 are offered under OSS licenses, and the majority of the remaining software is freely available to academic and non-profit members. For the very limited number of programs with restrictions imposed by the author or owner of the software, member laboratories must demonstrate license compliance before the application is installed.

This approach allows SBGrid to accommodate a variety of end-user types while complying with licensing terms of each program. For example, academic and non-profit members can access the entire collection by default (excluding applications with custom restrictions). For-profit members can access all OSS programs and any applications that permit free commercial use; however, they must provide SBGrid with proof of a valid license to access programs that have separate for-profit licensing agreements. Members with shared or mixed-use research environments, such as synchrotron beamlines, who often host both non-profit and for-profit users, are provided with multiple program trees each containing the appropriate subset of applications. These mixed-use facilities are then required to implement procedures for assuring that each category of user only has access to the appropriate program tree.

The discovery of new applications is enhanced within SBGrid by comprehensive inclusion and continuous addition of new software based on aggregated community knowledge. The formation of a community of active and involved researchers and scientist software developers around the collaboration also makes possible the collective exploitation of research computing resources that might otherwise be beyond the means of any single member or institution (such as the US cyber-infrastructure for large-scale computing: see Box 2).

For developers, distributing software through collaborations like SBGrid relieves the significant burdens associated with the distribution of their programs and the provision of end-user support, thereby freeing them to focus on new research and scientific communication with end-users rather than on technical support issues. Program creators are assured that updates, improvements, bug-fixes and other development-side maintenance modifications are rapidly deployed, and developers are apprised of which research groups have access to their software, giving them information to more easily track citations and assess scientific impact for grant and funding purposes. Furthermore, popular scientific programs that the original creator may no longer be able and/or willing to actively support or distribute may be offered and supported by the collaboration, thereby extending the lifetime of useful research software and maximizing return on research investment.

For institutions, collaborations like SBGrid offer a simple and cost-effective solution to providing domain-specific, up-to-date research computing resources for specialized scientific fields independent of in-house IT and research computing expertise. Thus, in an era of shrinking IT budgets, difficulty hiring and retaining experienced staff and continually expanding demands on IT resources, initiatives like SBGrid extend the range and quality of software support while allowing local research computing departments to focus on core competencies and delivering common, foundational IT services (see Figure 2).

## Generalizability of the SBGrid model

The community-based model established by SBGrid could offer benefits to other areas of research that are reliant on computing and scientific software. The basic requirements for replicating an SBGrid-like collaboration are: (i) a community of researchers utilizing common or overlapping research computing tools and methods; (ii) a set of useful research computing resources (such as scientist-created software or advanced computing facilities) whose acquisition, use or exploitation presents significant barriers to access. The ‘start-up’ phase of an SBGrid-style collaboration would also benefit from a cohort of research laboratories willing to provide an initial investment in foundational resources (e.g. salaries, facilities, etc). Alternatively, start-up could be facilitated through grant support from funding agencies, before shifting fully to a self-sustaining fee-for-use model. In the case of SBGrid the required technical elements are relatively modest (see Box 2) and are based on widely available and established technologies flexible enough to accommodate a broad range of research computing needs.

Critical to the long-term direction and success of SBGrid, and likely to other similar initiatives, are its financial support and governance models. On-going SBGrid operations are funded through yearly membership fees from participating research groups. Reliance on membership fees promotes the sustainability of the collaboration, independent of the typical five-year funding cycle, and helps assure SBGrid management remain responsive to community needs. Exploitation of new resources and exploratory development (such as the SBGrid Science Portal: see Box 2) undertaken by the collaboration are typically funded through competitive grant applications to funding agencies.

Of equal importance is the direction and governance of SBGrid by active researchers in structural biology. First-hand knowledge of current methods and practices, frequent contact with leading researchers and developers, and direct involvement in its community enables the custodians of SBGrid to effectively identify and administer new resources and provide appropriate and useful services to users.

More so than the technical details of compiling, deploying and maintaining a collection of software, these tenets of self-sustaining user-supported operations and administration and governance by active researchers in the field are key to long-term success of community-based collaborations.

## Conclusions

Collaborations like SBGrid provide a self-sustaining and community-responsive platform to address current and future challenges in research computing. As dependence on computational tools and techniques continue to increase in every field of scientific endeavour, the burdens of supporting research computing for individual researchers, research groups and institutions will also grow. SBGrid is an example of a successful community-based research computing initiative in the field of structural biology. Through the aggregation of resources, pooling of expertise and sharing of costs among its many members, SBGrid is able to lower barriers of entry and expand access to research computing. The SBGrid model is also readily generalizable to other scientific fields of study that rely on research computing, and would be likely to yield similar benefits.
